# Defect‐Rich Heterogeneous MoS_2_/NiS_2_ Nanosheets Electrocatalysts for Efficient Overall Water Splitting

**DOI:** 10.1002/advs.201900246

**Published:** 2019-05-20

**Authors:** Jinghuang Lin, Pengcheng Wang, Haohan Wang, Chun Li, Xiaoqing Si, Junlei Qi, Jian Cao, Zhengxiang Zhong, Weidong Fei, Jicai Feng

**Affiliations:** ^1^ State Key Laboratory of Advanced Welding and Joining Harbin Institute of Technology Harbin 150001 China; ^2^ School of Materials Science and Engineering Harbin Institute of Technology Harbin 150001 China; ^3^ MIIT Key Laboratory of Critical Materials Technology for New Energy Conversion and Storage State Key Laboratory of Urban Water Resource and Environment School of Chemistry and Chemical Engineering Harbin Institute of Technology Harbin 150001 China

**Keywords:** defects, free‐standing, heterointerfaces, metal sulfides, overall water splitting

## Abstract

Designing and constructing bifunctional electrocatalysts is vital for water splitting. Particularly, the rational interface engineering can effectively modify the active sites and promote the electronic transfer, leading to the improved splitting efficiency. Herein, free‐standing and defect‐rich heterogeneous MoS_2_/NiS_2_ nanosheets for overall water splitting are designed. The abundant heterogeneous interfaces in MoS_2_/NiS_2_ can not only provide rich electroactive sites but also facilitate the electron transfer, which further cooperate synergistically toward electrocatalytic reactions. Consequently, the optimal MoS_2_/NiS_2_ nanosheets show the enhanced electrocatalytic performances as bifunctional electrocatalysts for overall water splitting. This study may open up a new route for rationally constructing heterogeneous interfaces to maximize their electrochemical performances, which may help to accelerate the development of nonprecious electrocatalysts for overall water splitting.

The electrolysis of water offers a promising solution to produce clean and renewable hydrogen fuel supply.^1^ Electrocatalytic water splitting involving two half reactions, hydrogen evolution reaction (HER) and oxygen evolution reaction (OER), requires highly efficient electrocatalysts such as Pt for HER and RuO_2_ or IrO_2_ for OER to lower the activation barrier and boost the reaction process.^2^, ^3^ However, the high cost and scarcity of these noble electrocatalysts seriously limited their wide application.^4^ In view of the above situation, tremendous effort has been devoted to develop nonprecious yet efficient catalysts for OER and HER simultaneously.^5^, ^6^

Among these potential candidates, transition metal sulfides (TMSs), such as MoS_2_, CoS_2_, and NiS_2_, have drawn extensive attention, due to their considerable electrocatalytic performances.^7^, ^8^ However, the limited electroactive sites and insufficient stability of these TMSs seriously restricted the improved electrocatalytic activity.^9^, ^10^ Element doping would be an effective method to improve the catalytic activities, such as doping Ni atoms in MoS_2_.^1^, ^7^ Considering that surfaces or interfaces play the key role in electrochemical reactions, the morphology, surface defects or interfaces, and electrical structures are the key factors on the electrocatalytic performances of efficient catalysts.^11^, ^12^, ^13^, ^14^, ^15^ As for tuning the morphology, constructing 2D TMS nanosheets could generate abundant electroactive sites because of inherent large specific surface and rich active edges.^16^, ^17^ In particular, in situ growing nanosheet nanostructures on conductive substrates, such as Ni foam, carbon cloth, and stainless steel, could supply the efficient pathways for charge transport and provide open channels for rapid release of gas bubbles during OER or HER process.^18^, ^19^, ^20^ Further, interface modification could be another effective approach to engineering the physical or chemical properties of electrocatalysts.^21^, ^22^ The interface engineering could be beneficial to enrich the active sites and promote the electronic transfer, and thus boost the sluggish water splitting efficiency.^23^, ^24^ Additionally, owing to the different chemical reactivity, there are abundant interior defects in bimetal sulfide hybrids.^25^, ^26^ Undoubtedly, the defects in bimetal sulfide hybrids have a significant impact on electrical behavior, and thus modify the electrocatalytic performances.^25^, ^26^ Consequently, it is highly desirable to design and construct defected heterogeneous nanosheets with abundant electroactive sites directly on conductive substrates for overall water splitting.

Herein, we successfully construct defect‐rich heterogeneous MoS_2_/NiS_2_ nanosheets directly on carbon cloth and investigate the influence of interface configuration on the electrocatalytic performances. The defect‐rich interfaces with disordered structure are confirmed by the high‐resolution transmission electron microscopy (HRTEM), and X‐ray photoelectron spectroscopy (XPS) further evidences the strengthened interfacial effects between MoS_2_ and NiS_2_. Consequently, the optimal MoS_2_/NiS_2_ nanosheets present low overpotentials of 62 and 278 mV at 10 mA cm^−2^ for HER and OER, respectively. Further, for overall water splitting, the optimal MoS_2_/NiS_2_ nanosheets exhibit a voltage of 1.59 V at 10 mA cm^−2^ as well as good stability.

The fabrication of defect‐rich heterogeneous MoS_2_/NiS_2_ nanosheets involves two steps, as schematically shown in **Figure** **1**. First, Ni‐Mo precursors were synthesized on carbon cloth by the hydrothermal process. Second, MoS_2_/NiS_2_ nanosheets were synthesized by annealing the Ni‐Mo precursors with sublimed sulfur in Ar atmosphere at 400 °C for 1 h. And different MoS_2_/NiS_2_ nanosheets are prepared by finely controlling the amount of sublimed sulfur (50, 100, 200, and 400 mg), which is briefly named as MoS_2_/NiS_2_‐1, 2, 3, and 4. For comparison, pure NiS_2_ nanosheets also are prepared by the similar process and more detailed information could be found in Supporting Information. And pure NiMoO_4_ nanosheets are also synthesized by annealing Ni‐Mo precursors in air.

**Figure 1:**
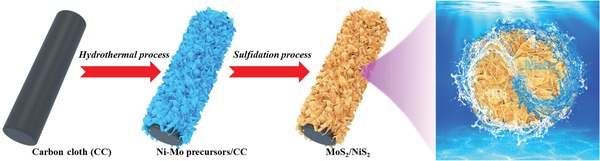
Schematic illustration for the formation of defect‐rich heterogeneous MoS_2_/NiS_2_ nanosheets.

The crystal phases of obtained samples are investigated by X‐ray diffraction (XRD) analysis, as shown in **Figure** **2**a. Evidently, all diffraction peaks except for the peak of carbon cloth could be well indexed to NiS_2_ (Joint Committee on Powder Diffraction Standards (JCPDS) Card No. 11‐0099)^6^ and MoS_2_ (JCPDS Card No. 37‐1492),^12^ suggesting the presence of metal sulfides. The morphologies of obtained samples are characterized by scanning electron microscopy (SEM), as shown in Figure 2b. A porous, discontinuous, and loose surface could be found in MoS_2_/NiS_2_‐3, while a continuous and compact surface could be observed for MoS_2_/NiS_2_‐1 (Figure S3a, Supporting Information). And with excessive sulfur source, the MoS_2_/NiS_2_‐4 nanosheets have been seriously etched and more porous nanosheets could be formed (Figure S3d, Supporting Information). From the SEM images in Figure 2b and Figure S3 in the Supporting Information, it can be found that various morphologies and structures of MoS_2_/NiS_2_ nanosheets could be prepared by finely controlling the amount of sublimed sulfur. Again, the TEM images in Figure 2c confirm that MoS_2_/NiS_2_‐3 nanosheets possess the amounts of nanoholes, which could provide more electroactive sites for catalytic reactions. The Brunauer–Emmett–Teller specific surface area of MoS_2_/NiS_2_‐3 nanosheets was calculated as 56.6 m^2^ g^−1^, again suggesting the rich active sites for electrochemical reactions. In the HRTEM images (Figure 2d–g), the lattice fringe of 0.62 nm belong to the (002) lattice plane of MoS_2_, while the lattice distances of 0.28 nm are indexed to (200) plane of NiS_2_. More importantly, clear heterointerfaces derived from the mismatch of MoS_2_ and NiS_2_ in Figure 2e,g could result in rich defects and disordered structure. Such heterointerfaces in obtained sulfides are beneficial for promoting HER performances, and it will be discussed in the following sections. Further, the energy dispersive X‐ray spectroscopy mapping results (Figure 2h) show the homogeneously distribution of Ni, Mo, and S elements in MoS_2_/NiS_2_‐3 nanosheets.

**Figure 2:**
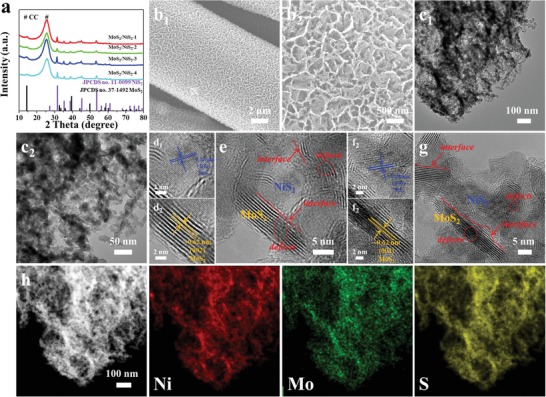
a) XRD patterns of obtained samples. b) SEM images of MoS_2_/NiS_2_‐3 nanosheets. c–g) TEM and HRTEM images of MoS_2_/NiS_2_‐3 nanosheets. h) The corresponding element mappings in MoS_2_/NiS_2_‐3 nanosheets.

Since electrochemical reactions mainly occur on the surfaces or at the interfaces of electrocatalysts,^11^, ^12^, ^13^ it is vital to investigate the surface states of obtained samples by Raman and XPS measurements. As shown in **Figure** **3**a, the peaks at about 294, 375, and 404 cm^−1^ belong to E_1g,_ E^1^_2g_, and A_1g_ modes of 2H‐MoS_2_, and the peak 345 cm^−1^ is from the modes of the Ni‐S.^8^, ^27^, ^28^, ^29^ More importantly, the obvious three peaks in 800–1000 cm^−1^ could be ascribed to the molecular structure of Mo_3_S_13_ that existing the edge sites of MoS_2_, and further suggest rich under‐coordinated Mo‐S edge sites in MoS_2_/NiS_2_‐3 nanosheets.^8^, ^28^, ^29^ As for Ni 2p in Figure 3b, two obvious peaks at 872.9 and 855.4 eV are attributed to the Ni 2p_1/2_ and Ni 2p_3/2_, as well two broad satellite peaks, demonstrating the presence of Ni^2+^.^30^ Compared to pure NiS_2_, two peaks of Ni 2p_1/2_ and Ni 2p_3/2_ in MoS_2_/NiS_2_‐3 are slightly shifted to higher binding energies (about 0.5 eV). It suggests the strong electronic interactions between NiS_2_ and MoS_2_ domains through established heterogeneous interfaces.^31^, ^32^ As for Mo 3d in Figure 3c, the peaks at about 232.4 and 229.1 eV are corresponded to Mo 3d_3/2_ and Mo 3d_5/2_, suggesting the presence Mo^4+^.^8^ And the nearby peak at about 226.2 eV from S 2s suggests the formation of Ni‐S and Mo‐S bindings.^8^, ^33^ For S 2p spectra in obtained samples (Figure 3d), it can be deconvoluted into three peaks corresponding to the S 2p_3/2_, S 2p_1/2_ for Mo‐S bond and S 2p_1/2_ for Ni‐S bond, at as well as a peak from the oxidized species formed on the surface of metal sulfides.^3^ The S 2p_3/2_ peak is attributed to the typical metal‐S bond, while the S 2p_1/2_ corresponds to the sulfur with low coordination that is generally related to sulfur defects.^34^, ^35^ It suggests the presence of terminal unsaturated S atoms on Mo‐S and Ni‐S sites, which may be beneficial for HER performances.^35^, ^36^ Based on SEM, TEM, Raman, and XPS analysis, as‐synthesized MoS_2_/NiS_2_ nanosheets are proved to possess porous nanostructure and defected interface for rich active sites and are expected to achieve excellent electrocatalytic performances.

**Figure 3:**
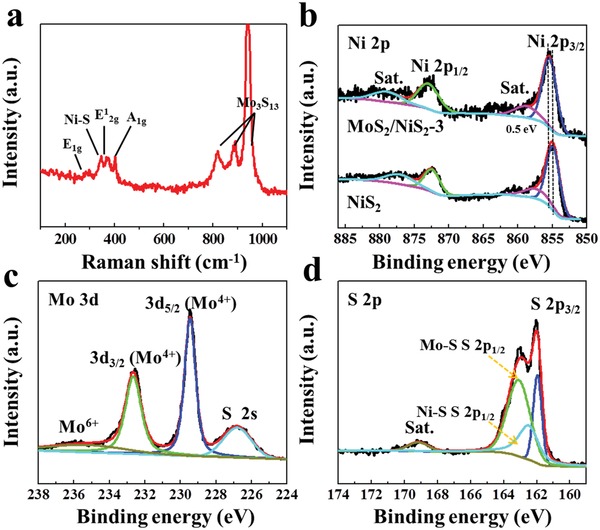
a) Raman spectrum of MoS_2_/NiS_2_‐3 nanosheets. High‐resolution XPS profiles of b) Ni 2p of MoS_2_/NiS_2_‐3 and NiS_2_, c) Mo 3d, and d) S 2p in MoS_2_/NiS_2_‐3 nanosheets.

The electrocatalytic HER and OER activities of heterogeneous MoS_2_/NiS_2_ nanosheets were measured in a three‐electrode configuration using 1 m potassium hydroxide (KOH) as electrolyte. For comparison, the performance of the commercial Pt/C, RuO_2_ and NiS_2_ were also measured. **Figure** **4**a presents the polarization curves of the samples with iR correction. As for MoS_2_/NiS_2_‐3 nanosheets, the current density rapidly increases with the increased potential, demonstrating the remarkable HER performances. The MoS_2_/NiS_2_‐3 nanosheets delivered an overpotential of 62, 108, and 131 mV at the current densities of 10, 50, and 100 mA cm^−2^, superior to those of other counterparts, and comparable with Pt/C. As further compared with the current noble‐metal‐free catalysts (Table S1, Supporting Information), our MoS_2_/NiS_2_‐3 nanosheets show the competitive electrocatalytic performances. When compared the HER performances of MoS_2_/NiS_2_ and pure NiS_2_, the obvious improvement suggests the synergy and mutual interaction between MoS_2_ and NiS_2_. In addition, we also prepared different samples by simply changing the annealing temperature, and the sublimed sulfur was fixed at 200 mg. As shown in Figures S6–S8 in the Supporting Information, MoS_2_/NiS_2_ obtained at 400 °C possesses the richest sulfur defects and shows the best HER and OER performances. These results demonstrate to some extent, sulfur defects could provide rich active sites and accelerate electron/mass transfer, resulting in improved catalytic performances.

**Figure 4:**
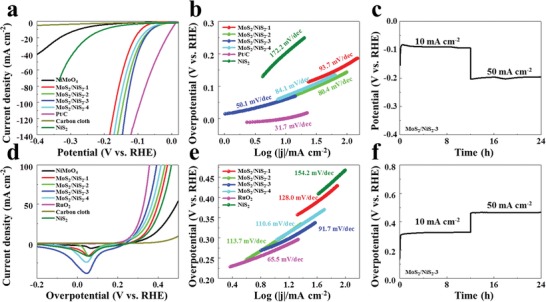
a) Polarization curves and b) the corresponding Tafel plots of obtained sample for HER. c) Chronopotentiometric curve of HER for MoS_2_/NiS_2_‐3 nanosheets. d) Polarization curves and e) the corresponding Tafel plots of obtained sample for OER. f) Chronopotentiometric curve of OER for MoS_2_/NiS_2_‐3 nanosheets.

To investigate the HER kinetic mechanism, the Tafel plots calculated from the polarization curves are shown in Figure 4b. The Tafel slop of MoS_2_/NiS_2_‐3 nanosheets (50.1 mV dec^−1^) is smaller than pure NiS_2_ and other MoS_2_/NiS_2_ samples, while it is close to that of Pt/C (31.7 mV dec^−1^). According to previous researches,^37^, ^38^ HER in KOH solution involves in three principal steps: i) Volmer reaction (Tafel slope of about 120 mV dec^−1^), ii) Heyrovsky reaction (Tafel slope of about 40 mV dec^−1^), and iii) Tafel reaction (Tafel slope of about 30 mV dec^−1^). Such a Tafel slope of the MoS_2_/NiS_2_ nanosheet (50.1 mV dec^−1^) suggests the HER reaction follows the Volmer–Heyrovsky mechanism (H_2_O + H_ads_ + e^−^ → H_2_ + OH^−^), where H_ads_ presents the H atom on an active site. Compared with pure NiS_2_ (172.2 mV dec^−1^), the obviously decreased Tafel slope of MoS_2_/NiS_2_‐3 also confirms the enhanced Volmer step in HER kinetics.^8^, ^30^ From the XPS result in Figure 3b, it could be inferred that the electron density around Ni is reduced in MoS_2_/NiS_2_‐3, which could provide sufficient empty d orbitals to improve the binding ability with H atoms.^31^, ^39^ Therefore, the improved H binding would facilitate the Volmer step, leading to the obviously decreased Tafel slope and enhanced HER performances of MoS_2_/NiS_2_. Figure S9a shows the chronopotentiometric curve for MoS_2_/NiS_2_‐3 electrode at various current densities from 10 to 190 mA cm^−2^. The potential shows no obvious changes in every step, indicating the good conductivity, excellent mass transport, and mechanical properties of MoS_2_/NiS_2_‐3 electrode in HER tests. In the long‐time stability test (Figure 4c), the MoS_2_/NiS_2_‐3 electrode maintains a stable HER activity at different current densities ranging from 10 to 50 mA cm^−2^. In addition, SEM and XRD results in Figure S10 in the Supporting Information demonstrate that the nanosheet structure and crystallinity of MoS_2_/NiS_2_‐3 are well retained after the HER stability measurement. Consequently, the good HER performances could be attributed to the strengthened interfacial effects between MoS_2_ and NiS_2_ with multilevel interfaces. Further, the rich electroactive sites by structure fine tuning also make great contribution to the good HER performances.

Since OER is another key role for overall water splitting, the OER performances of these samples are further characterized. As shown in Figure 4d, the polarization curve of MoS_2_/NiS_2_‐3 shows the remarkably improved OER activity with low overpotentials of 278, 352, and 393 mV at 10, 50, and 100 mA cm^−2^. Further, the OER performances of MoS_2_/NiS_2_‐3 are competitive among the current noble‐metal‐free catalysts (Table S2, Supporting Information). As shown in Figure 4e, the Tafel slope for MoS_2_/NiS_2_‐3 is 91.7 mV dec^−1^, which is lower than that of pure NiS_2_ (154.2 mV dec^−1^) and is close to that of RuO_2_ (65.5 mV dec^−1^). It indicates that the MoS_2_/NiS_2_‐3 proceeds a faster OER kinetic.^40^ The chronopotentiometric curve of MoS_2_/NiS_2_‐3 electrode in Figure S9b in the Supporting Information shows no obvious changes in every step, suggesting the good conductivity, excellent mass transport, and mechanical properties in OER tests. Further, we also compared the electrochemical sensitive area by measuring the double‐layer capacitance (*C*_dl_) of these samples in Figure S9c in the Supporting Information. The *C*_dl_ of 6.32 mF cm^−2^ on MoS_2_/NiS_2_‐3 is much higher than that of pure NiS_2_ (2.70 mF cm^−2^). Consequently, the higher *C*_dl_ value demonstrates the more efficient mass and charge transport capability on MoS_2_/NiS_2_‐3 for OER.^41^ Further, the Nyquist plots (Figure S9d, Supporting Information) show that the MoS_2_/NiS_2_‐3 possesses the lowest transfer resistance, revealing the fastest electron transfer kinetics.^40^, ^41^ To investigate the stability for OER, a long‐time chronopotentiometry measurement was carried out at 10 and 50 mA cm^−2^. As shown in Figure 4f, no obvious degradation could be found, suggesting the good stability.

In order to get insight the reaction mechanism for OER, the MoS_2_/NiS_2_‐3 after long‐term OER tests was characterized by SEM, XRD, Raman, and XPS (Figure S12, Supporting Information). MoS_2_/NiS_2_‐3 after long‐term OER tests maintained the nanosheet morphology with a rougher and thicker surface, as shown in Figure S12a in the Supporting Information. XRD pattern in Figure S12b in the Supporting Information shows that the new phase of Ni(OH)_2_·0.75H_2_O is formed after OER. It suggests that the Mo content in MoS_2_/NiS_2_‐3 is greatly reduced after long‐term OER tests. The Raman result in Figure S12c in the Supporting Information also shows the 471 and 558 cm^−1^ peaks from the presence of NiOOH.^42^ As for Ni 2p in Figure S12d in the Supporting Information, the energy separation of 17.6 eV demonstrate the presence of Ni^3+^.^43^, ^44^ In other word, it suggests that the surface Ni^2+^ was oxidized after continuous electrochemical tests. As in Figure S12e in the Supporting Information, the intensity of S 2p has been greatly reduced after OER stability tests, suggesting the loss of S element. And the intensity of Mo 3d (Figure S12f, Supporting Information) also suggests that Mo content is reduced after long‐term OER tests. With respect to O 1s (Figure S12g, Supporting Information), the peaks at 530.4 and 532.3 eV correspond to the lattice oxygen and surface hydroxyls.^45^ It suggests that nickel oxides/hydroxides are formed during OER process. According to previous researches,^45^, ^46^, ^47^, ^48^, ^49^ metal sulfides could be formed corresponding oxides/hydroxides during OER process and the formed oxides/hydroxides are known as the electrocatalytically active phases.

To illustrate the effect of the interface between MoS_2_ and NiS_2_, we also we synthesized pure MoS_2_ nanosheets on carbon cloth (see Figures S13 and S14, Supporting Information). Then, we scratched the MoS_2_ and NiS_2_ powders from the carbon cloth, then mechanically mixed MoS_2_ and NiS_2_ with the atomic ratio of Mo:Ni = 1:1 (denoted as MoS_2_‐NiS_2_). The mechanically mixed MoS_2_‐NiS_2_ powders were also prepared on carbon cloth for tests. As shown in Figure S15 in the Supporting Information, it can be found that MoS_2_/NiS_2_ shows much better HER and OER performances than that of mechanically mixed MoS_2_‐NiS_2_ sample. And according to previous researches,^50^, ^51^ interface engineering could be beneficial to accelerate the HER and OER kinetics by modifying the chemisorption. Owing to the strong electronic interactions and synergistic effects, the formed interfaces between two active materials could reconstruct more active centers for catalytic reactions.^50^, ^52^, ^53^ Consequently, these results further demonstrate the effect of the interface, which could enrich the active sites and promote the electronic transfer, and thus boost the sluggish water splitting efficiency.

Finally, the optimum MoS_2_/NiS_2_ was further used as bifunctional electrocatalyst for overall water splitting. As shown in **Figure** **5**a, the device affords a current density of 10 mA cm^−2^ with a cell voltage of 1.59 V. As shown in Figure 5b, the measured voltage is slightly larger than calculated voltage in different current densities, which may be due to the difference in the testing system. As shown in Figure 5c, the low cell voltage (1.59 V at 10 mA cm^−2^) for MoS_2_/NiS_2_ is competitive with recently reported bifunctional electrocatalysts, such as NiS‐NiS_2_ (1.58 V at 10 mA cm^−2^),^54^ Ni_0.33_Co_0.67_S_2_||NiCo_2_O_4_ (1.69 V at 10 mA cm^−2^),^55^ NiS‐Ni_2_P_2_S_6_ (1.64 V at 10 mA cm^−2^),^56^ MoS_2_/NiS (1.64 V at 10 mA cm^−2^),^52^ MoS_2_/NiS (1.61 V at 10 mA cm^−2^),^57^ NiS/Ni_2_P (1.67 V at 10 mA cm^−2^),^58^ NiS/NiS_2_ (1.62 V at 10 mA cm^−2^),^59^ Ni‐Co‐P (1.62 V at 10 mA cm^−2^),^60^ Co_3_O_4_@MoS_2_ (1.59 V at 10 mA cm^−2^),^61^ and MoS_2_‐CoOOH (1.60 V at 10 mA cm^−2^).^62^ To investigate the faradaic efficiency, the amounts of H_2_ and O_2_ produced during water splitting were measured in Figure S16 in the Supporting Information. After comparing the measured and calculated gas amounts, the faradaic efficiency for MoS_2_/NiS_2_ is closed to 100%. Further, Figure 5d shows that MoS_2_/NiS_2_||MoS_2_/NiS_2_ exhibits unnoticeable deterioration at 10 and 50 mA cm^−2^, suggesting good stability during overall water splitting.

**Figure 5:**
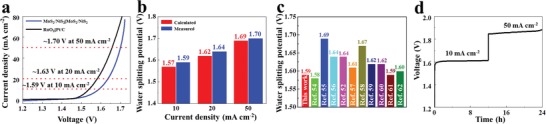
a) Polarization curves of optimal MoS_2_/NiS_2_ nanosheets and RuO_2_//Pt/C for overall water splitting. b) The comparison of calculated and measured water splitting potential. c) The comparison of overall water splitting performances between optimal MoS_2_/NiS_2_ nanosheets and other eletrocatalysts in reported literature. d) Chronopotentiometric curve of water electrolysis for optimal MoS_2_/NiS_2_ nanosheets.

Overall, the improved OER and HER performances of as‐synthesized MoS_2_/NiS_2_ nanosheets is attributed to following aspects: 1) nanosheet arrays directly on carbon cloth could make sure the efficient pathways for charge transport and open channels for rapid release of gas bubbles. 2) The nanoholes by finely controlling the amount of sublimed sulfur could provide rich electroactive sites for catalytic reactions. 3) The strengthened interfacial effects between MoS_2_ and NiS_2_ could effectively modify the electronic interactions, contributing the electrocatalytic activities. 4) The abundant heterogeneous interfaces in MoS_2_/NiS_2_ could not only provide rich electroactive sites but also facilitate the electron transfer. Consequently, optimal MoS_2_/NiS_2_ nanosheets show the enhanced electrocatalytic performances for HER, OER, and overall water splitting.

In summary, free‐standing and defect‐rich heterogeneous MoS_2_/NiS_2_ nanosheets are successfully designed and fabricated, which serves as bifunctional electrocatalysts for overall water splitting. The derived MoS_2_/NiS_2_ interface with rich defects and disordered structure could modify the electronic interactions and facilitate the electron, which could be beneficial for electrocatalytic reactions. Further, the binder‐free nanosheet arrays with rich nanoholes could also boost the HER and OER performances by providing rich electroactive sites and favoring the gas release from nanosheets. Consequently, working as both cathode and anode electrodes, the optimal MoS_2_/NiS_2_ nanosheets exhibit a voltage of 1.59 V at the current density of 10 mA cm^−2^, as well as good stability. Therefore, rational construct and comprehension of heterogeneous interfaces offer a promising alternative to nonprecious electrocatalysts for overall water splitting.

## Conflict of Interest

The authors declare no conflict of interest.

## Supporting information

Supplementary
