# Supplementary material for: Defect‐Rich Heterogeneous MoS2/NiS2 Nanosheets Electrocatalysts for Efficient Overall Water Splitting
Source: Adv Sci (Weinh). 2019 May 20;6(14):1900246. doi: 10.1002/advs.201900246 (PMC6661938; doi:10.1002/advs.201900246)
Supplement: Supplementary file 1 — Supplementary [file ADVS-6-1900246-s001.pdf]

Copyright WILEY-VCH Verlag GmbH & Co. KGaA, 69469 Weinheim, Germany, 2019.

ADVANCED SCIENCE
Open Access

# Supporting Information

for *Adv. Sci.,* DOI: 10.1002/advs.201900246

# Defect-Rich Heterogeneous $MoS_2$/$NiS_2$ Nanosheets Electrocatalysts for Efficient Overall Water Splitting

*Jinghuang Lin, Pengcheng Wang, Haohan Wang, Chun Li, Xiaoqing Si, Junlei Qi,* Jian Cao, Zhengxiang Zhong, Weidong Fei, and Jicai Feng*

**Supporting Information**

# Defect-rich heterogeneous $MoS_2$/$NiS_2$ nanosheets electrocatalysts for efficient overall water splitting

Jinghuang Lin, Pengcheng Wang, Haohan Wang, Chun Li, Xiaoqing Si, Junlei Qi*****,

Jian Cao, Zhengxiang Zhong, Weidong Fei and Jicai Feng

## 1. Experimental Section

***Fabrication of $MoS_2/NiS_2$ nanosheets:*** The commercial carbon cloth was treated with concentrated $HNO_3$,ethanol and deionized water several times, according to previous research.[1] Firstly, 10 mmol $Ni(NO_3)_2 \cdot 6H_2O$, 1.43 mmol $(NH_4)_6Mo_7O_{24} \cdot 4H_2O$ and 20 mmol hexamethylenetetramine were dissolved in 80 mL distilled water and stirred for 2 h. Then, the mixed solution with a piece of carbon cloth ($2 \times 5$ $cm^2$) was transferred to a 100 mL Teflon-lined stainless steel autoclave and heated to 150 $^oC$ for 12 h. The obtained Ni-Mo precursors were washed by distilled water and ethanol several times, and dried at 80 ℃ overnight. Secondly, sublimed sulfur and a piece of carbon cloth with Ni-Mo precursors were put at the upstream and downstream side of the tube furnace, then the furnace was heated to 400 $^oC$ for 1h with a ramp rate of 5 $^oC$ under Ar atmosphere to obtain $MoS_2/NiS_2$**.** To obtain different samples, various amounts of sublimed sulfur (50, 100, 200 and 400 mg) was used. For comparison, pure $NiS_2$ nansheets were synthesized by similar processes without Mo source, and the amount of sublimed sulfur is 200 mg. For comparison, Ni-Mo precursors were annealed in 400 $^oC$ for 2h in air to obtain $NiMoO_4$ samples.

**Sample characterization**

SEM (Helios Nanolab 600i), TEM (Tecnai G2 F30) and XRD (D8 Advance) were applied to investigate the nanostructure, morphology and crystalline phases of obtained samples. XPS (Thermo Fisher) and Raman (InVia-Reflex) were used to investigate the surface states. The Nitrogen adsorption/desorption isotherms were conducted at 77 K.

**Electrochemical measurements**

All electrochemical performances were measured in the electrochemical workstation

(CHI 760E and PARSTAT 4000A). The OER and HER properties were measured in a three-electrode system, using obtained samples, Hg/HgO and carbon rod as working electrode, reference electrode and counter electrode, respectively. All the potential was converted to RHE. The polarization curves were measured at 2 mV $s^{-1}$, and were compensated with iR-correction. Before OER and HER tests, all samples were cycled at 10 mV $s^{-1}$ until the stability of cyclic voltammetry (CV), then the data were collected. The overall water splitting was tested in the two-electrode system. The reference electrodes of Pt/C (20 wt%) and $RuO_2$ were also prepared on carbon cloth, and the prepared method was according to previously reported researches.[2,3]

**Reference**

1. L. An, J. Feng, Y. Zhang, R. Wang, H. Liu, G. Wang, F. Cheng and P. Xi, *Adv. Funct. Mater.* 2019, **29**, 1805298.

2. Z. Zhang, X. Ma and J. Tang, *J. Mater. Chem. A* 2018, **6**, 12361.

3. Z. Wang, H. Liu, R. Ge, X. Ren, J. Ren, D. Yang, L. Zhang and X. Sun, *ACS Catal.* 2018, **8**, 2236.

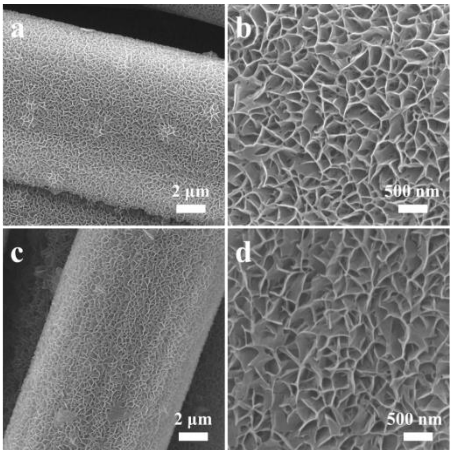


**Fig. S1** SEM images of (a,b) NiMo-precursor and (c,d) $NiMoO_4$.

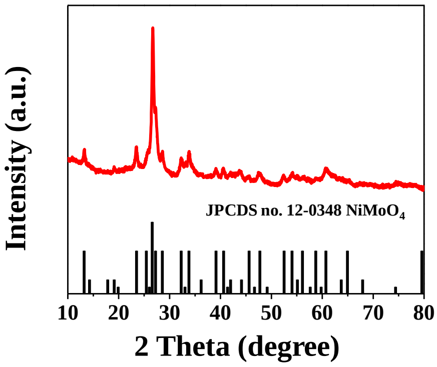


**Fig. S2** XRD pattern of $NiMoO_4$ scratched from the carbon cloth.

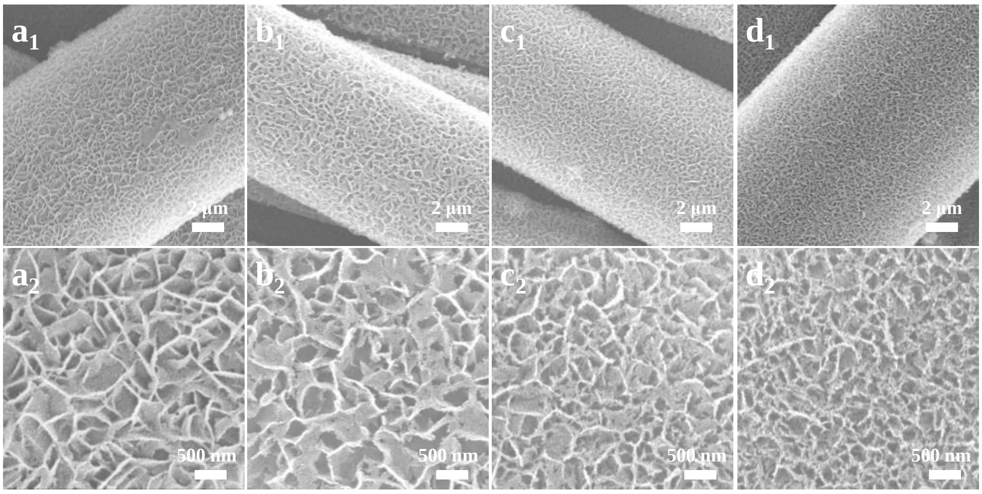


**Fig. S3** SEM images of (a) $MoS_2/NiS_2$-1, (b) $MoS_2/NiS_2$-2, (c) $MoS_2/NiS_2$-3 and (d) $MoS_2/NiS_2$-4.

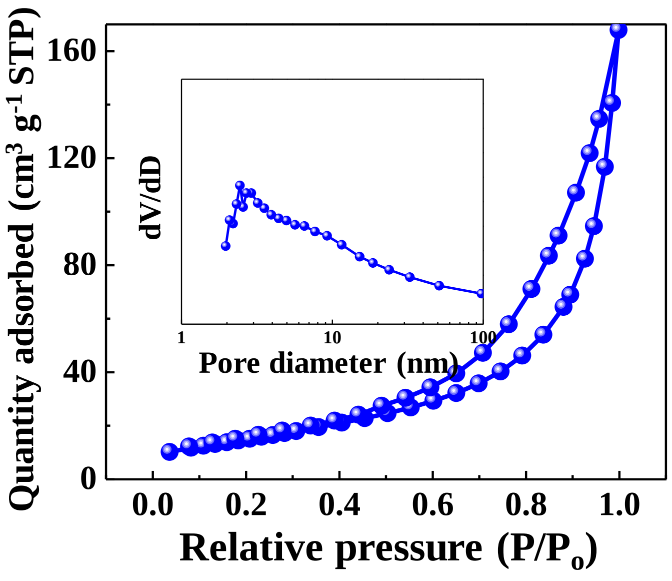


**Fig. S4** The $N_2$ adsorption/desorption isotherm of $MoS_2/NiS_2$-3, and the inset is the corresponding pore size distribution

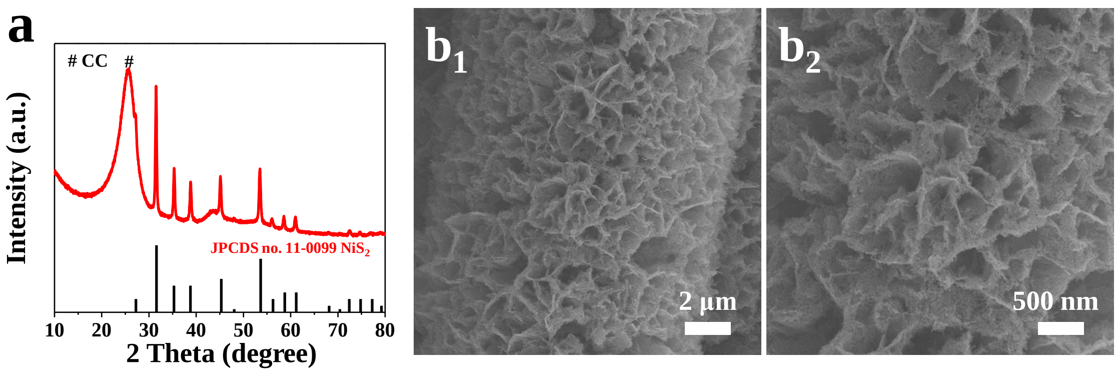


**Fig. S5** (a) XRD pattern and (b) SEM images of pure $NiS_2$ nanosheets on carbon cloth.

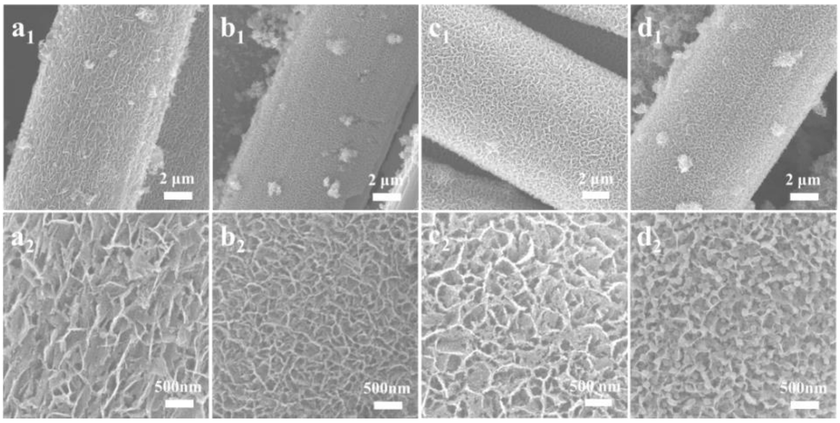


**Fig. S6** SEM images of (a) $MoS_2/NiS_2$-300, (b) $MoS_2/NiS_2$-350, (c) $MoS_2/NiS_2$-400 and (d) $MoS_2/NiS_2$-500.

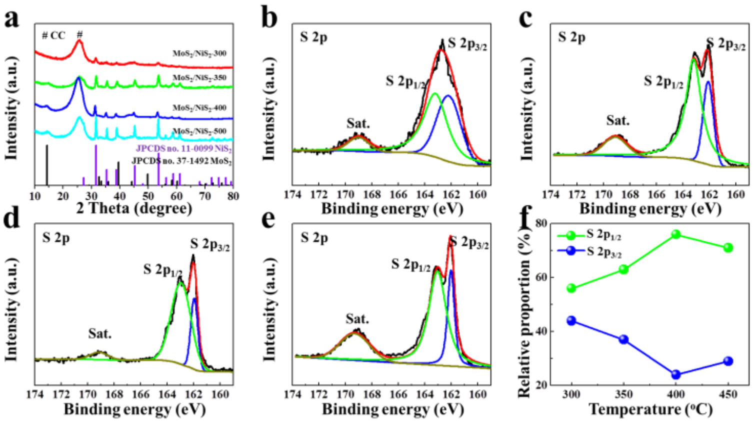


**Fig. S7** (a) XRD patterns of obtained samples under different vulcanization temperature. S 2p spectra of samples obtained by different vulcanization temperatures: (b) 300 °C, (c) 350 °C, (d) 400 °C and (e) 500 °C. (f) The relative proportion of S $2p_{1/2}$ and S $2p_{3/2}$ of obtained samples under different vulcanization temperature.

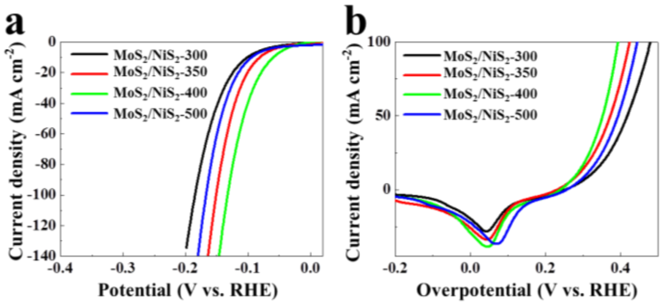


**Fig. S8** Polarization curves of $MoS_2/NiS_2$ obtained at different temperatures for (a) HER and (b) OER.

As for defects, we prepared different samples by simply changing the annealing temperature (300, 350, 400 and 500 $^{o}$C, denoted as $MoS_2/NiS_2$-300, 350, 400, 500), and the sublimed sulfur was fixed at 200 mg. As shown in **Fig. S6**, all samples maintain the nanosheet morphologies. As shown in **Fig. S7a**, all diffraction peaks except for the peak of carbon cloth could be well indexed to $NiS_2$ and $MoS_2$. To investigate the sulfur defects, we perform XPS analysis in **Fig. S7b-7f**. For S 2p in each sample, the peak at the lower binding energy is S $2p_{3/2}$ core level from metal-sulfur bonds,[1,2] while S $2p_{1/2}$ at the higher binding corresponds to the sulfur with low coordination that is generally related to sulfur vacancies.[3,4] Accordingly, the S $2p_{1/2}$/S $2p_{3/2}$ surface ratio of obtained samples is shown in **Fig. S7f**. It can be found that the more intensity of S $2p_{1/2}$ and less intensity of S $2p_{3/2}$ are observed, suggesting forming more sulfur defects. When the the annealing temperature reaches 400 $^{o}$C, the S $2p_{1/2}$/S $2p_{3/2}$ surface ratio is largest, suggesting the richest sulfur defects in obtained samples. We also tested the HER and OER performances of these obtained samples in **Fig. S8**. The LSV polarization curves show that $MoS_2/NiS_2$-400 exhibit an admirable alkaline HER and OER performances, which is much better than thoes of other counterparts. These results demonstrate that, to some extent, sulfur defects could provide rich active sites and accelerate electron/mass transfer, resulting in improved catalytic performances.

**Reference**


1. J. Xiao, L. Wan, S. Yang, F. Xiao and S. Wang, *Nano Lett.* 2014, **14**, 831.

2. H. Chen, J. Jiang, L. Zhang, H. Wan, T. Qi and D. Xia, *Nanoscale* 2013, **5**, 8879.

3. K. Andersson, M. Nyberg, H. Ogasawara, D. Nordlund, T. Kendelewicz, C. S. Doyle, G. E. Brown, L. G. M. Pettersson and A. Nilsson, *Phys. Rev. B* 2004, **70**, 195404.

4. F. Lu, M. Zhou, W. Li, Q. Weng, C. Li, Y. Xue, X. Jiang, X. Zeng, Y. Bando and D. Golberg, *Nano Energy* 2016, **26**, 313.

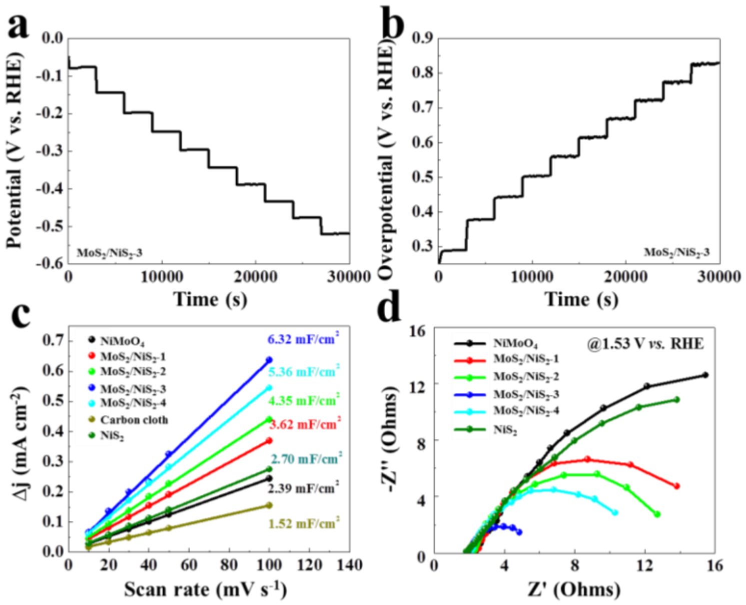


**Fig. S9** The chronopotentiometric curves of $MoS_2/NiS_2$-3 from 10 to 190 mA cm$^{-2}$ with an increment of 20 mA cm$^{-2}$ every 3000 s in (a) HER and (b) OER tests (without iR-correction). (c) The current density against scan rate of obtained samples. (d) The Nyquist plots of obtained samples at 1.53 V vs. RHE.

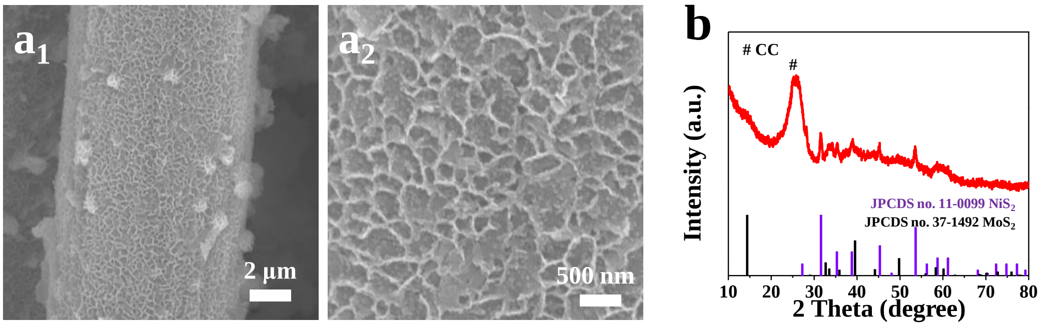


**Fig. S10** SEM images and XRD pattern of $MoS_2/NiS_2$-3 after long-term HER tests.

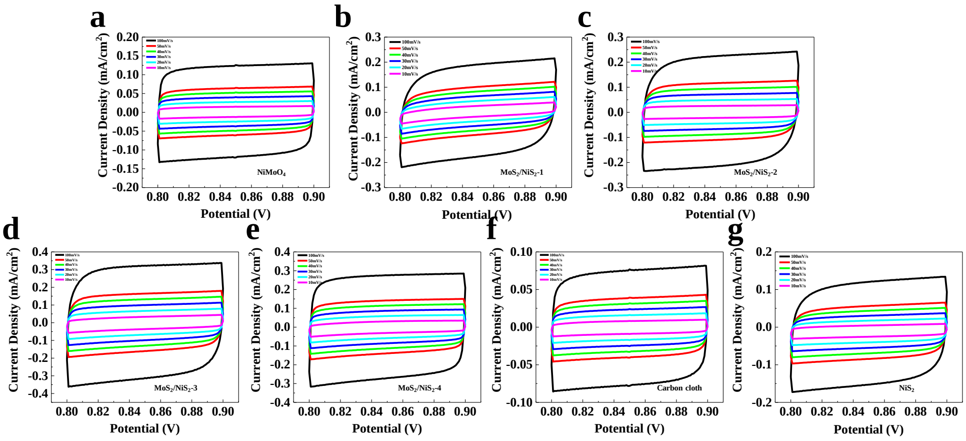


**Fig. S11** CV curves of obtained samples in the window of 0.80-0.90 V vs. RHE.

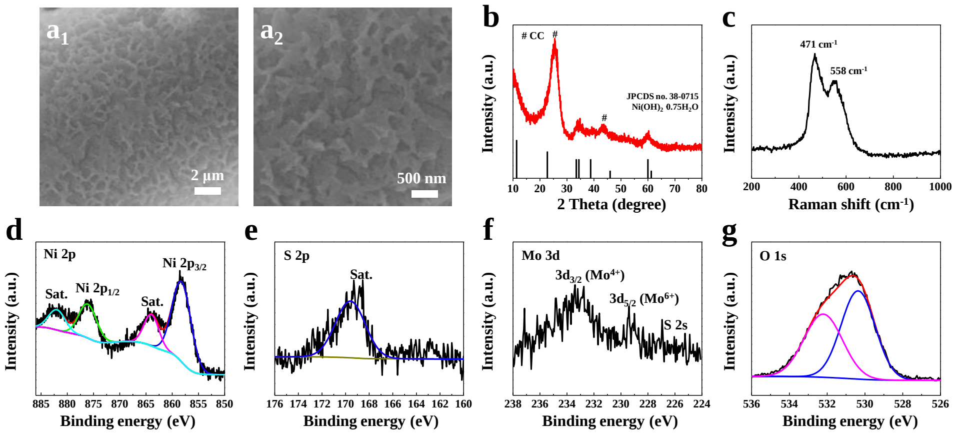


**Fig. S12** (a) SEM images, (b) XRD, (c) Raman spectrum and XPS (d) Ni 2p, (e) S 2p, (f) Mo 3d, (g) O 1s spectra of $MoS_2/NiS_2$-3 after long-term OER tests.

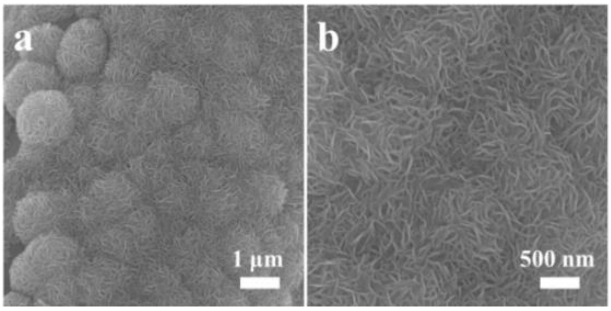


**Fig. S13** SEM images of $MoS_2$ on carbon cloth.

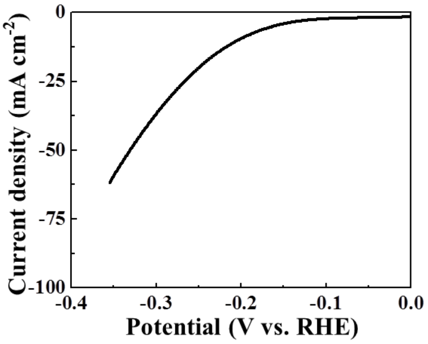


**Fig. S14** Polarization curves of MoS for HER.

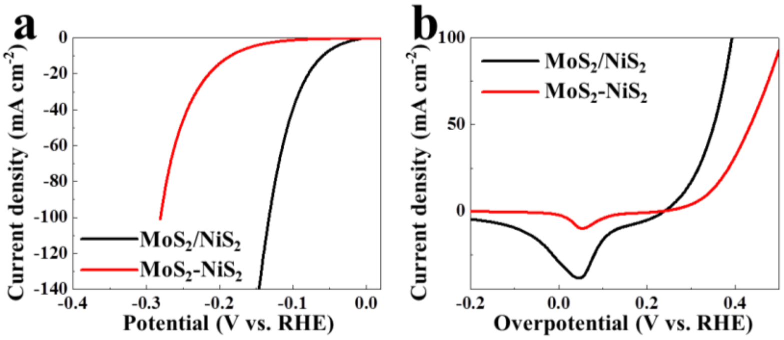


**Fig. S15** Polarization curves of $MoS_2$-$NiS_2$ and $MoS_2/NiS_2$ for (a) HER and (b) OER.

We also synthesized pure $MoS_2$ nanosheets on carbon cloth.[1] Then, we scratched the $MoS_2$ and $NiS_2$ powders from the carbon cloth, then mechanically mixed $MoS_2$ and $NiS_2$ with the atomic ratio of Mo:Ni =1:1 (denoted as $MoS_2$-$NiS_2$). The mechanically mixed $MoS_2$-$NiS_2$ powders with mass loading of 1.1 mg cm$^{-2}$ were also prepared on carbon cloth. Finally, we compared the HER and OER performances of $MoS_2$-$NiS_2$ and $MoS_2/NiS_2$. As shown in **Fig. S15**, it can be found that $MoS_2/NiS_2$ shows much better HER and OER performances than that of mechanically mixed $MoS_2$-$NiS_2$ sample. Consequently, these results further demonstrate the effect of the interface, which could enrich the active sites and promote the electronic transfer, and thus boost the sluggish water splitting efficiency.

**Reference:**

1. H. Zhang, L. Yu, T. Chen, W. Zhou and X. W. Lou, *Adv. Funct. Mater.* 2018, **28**, 1807086.

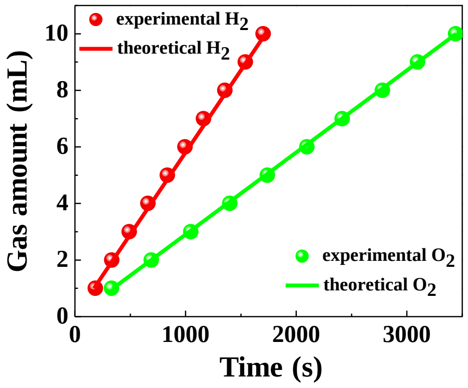


**Fig. S16** The theoretical and experimental gas amounts in different time for overall water splitting of optimal $MoS_2/NiS_2$ nanosheets.

**Table S1** Comparison of HER performances for $MoS_2/NiS_2$ nanosheets with previsoulsy reported electrocatalysts in the alkaline media.

| Electrocatalyst | Substrate | Overpotential (mV) | Tafel slope (mV dec$^{-1}$) | Ref. |
|---|---|---|---|---|
| $MoS_2/NiS_2$ | Carbon cloth | 62, 108, 131 at 10, 50, 100 mA cm$^{-2}$ | 50.1 | **This work** |
| CoP nanowire by oxygen plasma engraving | Carbon cloth | 180 at 100 mA cm$^{-2}$ | 42.8 | *Adv. Mater.* 2018, **30**, 1703322. |
| Cobalt Selenide | Co foil | 268 at 100 mA cm$^{-2}$ | 61.4 | *Adv. Energy Mater.* 2018, **8**, 1801926. |
| $Ni_2P/Fe_2P$ | Ti foil | 121, ~210 at 10, 100 mA cm$^{-2}$ | 67 | *Adv. Energy Mater.* 2018, **8**, 1800484 |
| $Ni_2P$-$Ni_3S_2$ | Ni foam | 80, ~175 at 10, 100 mA cm$^{-2}$ | 65 | *Nano Energy* 2018, **51**, 26. |
| $Co_5Mo_{1.0}P$ nanoshets | Ni foam | 173, 300 at 10, 100 mA cm$^{-2}$ | 190.1 | *Nano Energy* 2018, **45**, 448. |
| Partially oxidized Ni nanoparticles | Carbon nanofibers | 262, 295, 343, 371 at 10, 20, 50, 80 mA cm$^{-2}$ | 97.42 | *Nano Energy* 2018, **51**, 286 |
| Co-$Ni_3N$ | Carbon cloth | 194 at 10 mA cm$^{-2}$ | 156.0 | *Adv. Mater.* 2018, **30**, 1705516 |
| $CoNiPS_3$/C nanosheets | Glass carbon | 136 at 30 mA cm$^{-2}$ | 60 | *Adv. Mater.* 2018, **28**, 1805075 |
| Mo-doped $Ni_3S_2$ nano-rods | Ni foam | 180 at 100 mA cm$^{-2}$ | 72.9 | *J. Mater. Chem. A* 2017, **5**, 1595. |
| N-$Ni_3S_2$ | Ni foam | 110, ~240 at 10, 100 mA cm$^{-2}$ | - | *Adv. Mater.* 2017, **29**, 1701584. |
| $SrCo_{0.85}Fe_{0.1}P_{0.05}O_{3-\delta}$ nanofilm | Ni foam | 110 at 10 mA cm$^{-2}$ | 94 | *Adv. Mater.* 2018, **30**, 1804333 |
| NC/CuCo/CuCoOx nanowires arrays | Ni foam | 112, 190 at 10, 100 mA cm$^{-2}$ | 55 | *Adv. Funct. Mater.* 2018, **28**, 1704447 |
| $TiO_2@Co_9S_8$ core-branch arrays | Ni foam | 139, ~190 at 10, 50 mA cm$^{-2}$ | 65 | *Adv. Sci*. 2018, **5**, 1700772 |
| Fe-Ni@NC-CNTs | Glassy carbon dish | 202 at 10 mA cm$^{-2}$ | 113.7 | *Angew. Chem.* 2018, **130**, 9059 |
| Fe doped $Ni_3S_2$ | Ni foam | 47, 232 at10, 100 mA cm$^{-2}$ | 95 | *ACS Catal.* 2018, **8**, 5431. |

1. If not metioned specifically, all overpotentials were corrected with iR compensation. 2. If not metioned specifically, all electrocatalysts are directly synthesized on conductive substrates.

**Table S2** Comparison of OER performances for $MoS_2/NiS_2$ nanosheets with previsoulsy reported electrocatalysts in the alkaline media.

| Electrocatalyst | Substrate | Overpotential (mV) | Tafel slope (mV $dec^{-1}$) | Ref. |
|---|---|---|---|---|
| $MoS_2/NiS_2$ | Carbon cloth | 278, 352, 393 at 10, 50, 100 mA $cm^{-2}$ | 91.7 | **This work** |
| $NiCo_2P_2$/graphene quantum dot | Ti mesh | 400 at 100 mA $cm^{-2}$ | 65.9 | *Nano Energy* 2018, **48**, 284. |
| Partially oxidized Ni nanoparticles | Carbon nanofibers | 420, 470, 560, 630 at 10, 20, 40, 60 mA $cm^{-2}$ | 113.1 | *Nano Energy* 2018, **51**, 286 |
| Co-$Ni_3N$ | Carbon cloth | 307 at 10 mA $cm^{-2}$ | 57.0 | *Adv. Mater.* 2018, **30**, 1705516 |
| $CoNiPS_3$/C nanosheets | Glass carbon electrodes | 262 at 30 mA $cm^{-2}$ | 56.0 | *Adv. Mater.* 2018, **28**, 1805075 |
| $CoS_2$ nanotube | Carbon cloth | 276 at 10 mA $cm^{-2}$ | 81 | *Nanoscale Horiz.* 2017, **2**, 342--348 |
| $SrCo_{0.85}Fe_{0.1}P_{0.05}O_{3-\delta}$ nanofilm | Ni foam | 310 at 10 mA $cm^{-2}$ | 55 | *Adv. Mater.* 2018, **30**, 1804333 |
| NiO@Ni/$WS_2$ | Carbon cloth | 380 at 50 mA $cm^{-2}$ | 108.9 | *ACS Cent. Sci.* 2018, **4**, 112. |
| $Fe_2O_3$-CoP | Indium tin oxide | 302 at 10 mA $cm^{-2}$ | - | *J. Mater. Chem. A*, 2018, **6**, 4783. |
| Mo-CoOOH | Carbon cloth | 305, 365 at 10, 100 mA $cm^{-2}$ | 56 | *Nano Energy* 2018, **48**, 73. |
| Fe-Ni@NC-CNTs | Glassy carbon dish | 274 at 10 mA $cm^{-2}$ | 45.5 | *Angew. Chem.* 2018, **130**, 9059 |
| Mo-NiOOH | Ni foam | 390 at 100 mA $cm^{-2}$ | 68 | *Int. J. Hydrogen Energy* 2018, **43**, 12140. |
| NiCoP cone shaped nanowire | Ni foam | 370 at 100 mA $cm^{-2}$ | 116 | *J. Mater. Chem. A* 2017, **5**, 14828. |
| $Co_3O_4$-N-C frameworks | Glassy carbon | 324 at 10 mA $cm^{-2}$ | 69 | *Nano Energy* 2018, **48**, 600 |
| $Ni_3S_2$@$MoS_2$/FeOOH | Ni foam | 260 at 10 mA $cm^{-2}$ | 49 | *Appl. Catal. B* 2019, **244**, 1004. |
| $Fe_{0.09}Co_{0.13}$-$NiSe_2$ nanosheets | Carbon cloth | 251 at 10 mA $cm^{-2}$ | 63 | *Adv. Mater.* 2018, 30, 1802121 |

1.If not metioned specifically, all overpotentials were corrected with iR compensation. 2. If not metioned specifically, all electrocatalysts are directly synthesized on conductive substrates.
